# Differential Detection of Potentially Hazardous *Fusarium* Species in Wheat Grains by an Electronic Nose

**DOI:** 10.1371/journal.pone.0021026

**Published:** 2011-06-09

**Authors:** Jakob Eifler, Eugenio Martinelli, Marco Santonico, Rosamaria Capuano, Detlev Schild, Corrado Di Natale

**Affiliations:** 1 Department of Crop Sciences, Georg-August-Universität Göttingen, Göttingen, Germany; 2 Department of Neurophysiology and Cellular Biophysics, Georg-August-Universität Göttingen, Göttingen, Germany; 3 Department of Electronic Engineering, University of Rome, Rome, Italy; 4 Bernstein Focus of Neurotechnology, University of Göttingen, Göttingen, Germany; University of California , Merced, United States of America

## Abstract

Fungal infestation on wheat is an increasingly grave nutritional problem in many countries worldwide. *Fusarium* species are especially harmful pathogens due to their toxic metabolites. In this work we studied volatile compounds released by *F. cerealis, F. graminearum, F. culmorum* and *F. redolens* using SPME-GC/MS. By using an electronic nose we were able to differentiate between infected and non-infected wheat grains in the post-harvest chain. Our electronic nose was capable of distinguishing between four wheat Fusaria species with an accuracy higher than 80%.

## Introduction

*Fusarium* species, *F. graminearum* and *F. culmorum* in particluar, are widespread pathogens in all cereal growing areas worldwide and one of the most important genera of plant pathogenic fungi on earth [1], [2]. Toxic metabolites, especially the mycotoxin deoxynivalenol (DON, Vomitoxin) and zearalenone (ZEA) are produced from these genera [3].

In 2001 the WHO/FAO carried out a world-wide study comprising Argentina, Brazil, Canada, China, Finland, Germany, Italy, the Netherlands, Norway, Sweden, the United Kingdom, Uruguay and the USA. It was estimated therein that, on the average, 57% of the wheat samples analyzed (11.444) contained deoxynivalenol (DON) [4]. Results from random analysis in Germany indicate that only 29% of all cereal-based products are devoid of *Fusarium-*toxins [5]. The mycotoxin thus appears to spread to food products becoming a potential and presumably serious health risk to humans [6]. Thus, the FAO has issued guidelines and regulatory limits for *Fusarium* mycotoxins [7].

As a consequence, there is an absolute need for early and readily applicable methods to detect *Fusarium-*infected grain and to distinguish between relevant and harmless species. So far *Fusarium* metabolites have been detected and analyzed using GC-MS [8], LC-UV or LC-MS [9], TLC [10], fluorescence immunoassays [11], NIRS [12], HPLC-MS [13], ELISA [14], whereas whole fungi were detected using ELISA or PCR techniques [15]–[17]. While some of these approaches are able to detect specific species, they commonly lack quantitative analysis [18]. Others do not allow differentiating between different *Fusarium* species (ELISA) [17]. Quantitative PCR turned out to be the most precise method, albeit expensive and time-consuming [17]. However, all of the methods used so far are laboratory–based, and none of them allows the on-line detection and quantification, possibly in the field.

Fusaria have been shown to emanate a number of volatile compounds, specifically carbonyls, hydrocarbons, ketones, terpenes and complex mixtures of alcohols [19]–[24]. While these studies suggested a number of relevant compounds, none of them turned out to be a specific marker for any of the Fusaria. It thus seems to be the pattern of chemicals that is characteristic for any of the species. In the laboratory, such patterns would commonly be analyzed using standard analytical equipment, e.g., gas chromatography–mass spectrometry (GC–MS) [25]. A similar objective can be obtained by using an electronic nose [26], i.e., an array of solid-state sensors that are non-selectively sensitive to the relevant chemicals and the responses of which reflect the chemical information contained in the sample. Note that this detection scheme is in many respects similar to natural olfaction where hundreds of different receptors allow to distinguish among tens of thousands of different odors [27]. Electronic noses have been applied in different fields providing useful identification and classification of samples [28]. The identification of contaminated grains was attempted by several groups. These studies were based on various sensor technologies such as chemosensitive field effect transistors [29], conducting polymers [30], metal oxide semiconductors [31] and quartz microbalances [32].

To differentiate on-line between whole, dry wheat grains that were differentially contaminated by four *Fusarium* species, we here used an electronic nose based on an array of metalloporphyrin-coated quartz microbalances. The discrimination properties of this instrument were demonstrated in several applications to study food processes [33] and lung cancer diagnosis from breath analysis and medical diagnosis [34].

## Materials and Methods

### Samples

Grains from soft wheat (cv Isengrain, harvest season 2009, Germany) were used. The seeds were water-saturated for 24 hours to ensure rehydration and then autoclaved twice for 15 min at 121°C. For each sample 100 g sterilized kernels were inoculated with ten 0.5×0.5 cm^2^ slices of fungal mycelia derived from cultures grown on potato dextrose agar (PDA). Incubation was carried out for 5, 10 and 15 days at a relative humidity of 70% and at 27°C. Infected samples were dried to 13% moisture content and stored at 4°C to block further fungal growing. We used the fungus species *Fusarium graminearum*, *Fusarium culmorum, Fusarium cerealis* and *Fusarium redolens*. Sterilized, non-infected kernels incubated over 0, 5, 10 or 15 days as well as an untreated probe served as controls. The number of samples was chosen in respect of the sample variability artificially induced by inoculating microorganisms in homogeneous grain samples. Thus, the influence of the natural variability among grains, e.g., due to species variability and crop production is not taken into account here.

### Headspace generation

For both GC/MS and Enose measurements, grain samples (3 g) were enclosed in Teflon-sealed vials. Prior to the measurements, the samples were kept in a thermal bath for 30 min at constant temperature. The Enose experiments were carried out at a sample temperature of 30°C. GC/MS analyses were done at 30°C and 70°C. In addition, an empty vial was added as a reference air source.

### Electronic Nose analysis

The core of the electronic nose used in this paper consisted of an array of eight quartz microbalances (QMB), each being a quartz crystal resonator with mass-dependent eigenfrequency f. Slight mass changes (*Δ*m) of the quartz surface result in frequency changes (*Δ*f) of the electrical output signal of the oscillator circuit. The quantities *Δ*m and *Δ*f are linearly related to each other in the low-perturbation regime [35]. Our Enose consisted of QMB with a fundamental frequency of 20 MHz and a mass sensitivity in the order of a few nanograms. QMBs can be turned into chemical sensors by coating their surfaces with layers of chemically sensitive materials. In this work we coated the QMBs with layers of metalloporphyrins. Regarding their sensing properties metalloporphyrins host several interaction mechanisms from weak and non-selective Van der Waals forces to the more energetic and specific coordination of the central metal atom. The balance between these forces can be controlled by the nature of the porphyrins' peripheral group and the metal atom, so that metalloporphyrins with different sensitivities for volatile compounds can be obtained [36] and assembled to sensor arrays for electronic noses [37].

For the experiments of this paper, the grain samples were closed in a sealed vial with an inlet and an outlet. Vials were kept at a constant temperature in order to allow for a stable headspace composition. The headspace was extracted by a flow of ambient air, filtered through a CaCO3 bed. The flow was maintained constant at 7.5 ml/min by a peristaltic pump of the electronic nose. The filtered ambient air was also used to clean the sensors and to establish the reference signal. Sensor signals were calculated as the signal frequency shift, *Δ*f = fs−fa, with fs and fa being obtained from the sample headspace and filtered ambient air. Sensors were exposed to a sample for 60 s, followed by a 5 min cleaning and regeneration phase with reference air. All measurements were repeated three times.

### GC/MS analysis

GC/MS analysis were performed using a gas chromatograph (QP2010, Shimadzu, Japan) connected to a mass spectrometer. An EQUITY-5 capillary column (l, 30 m; i.d., 0.25 mm; 0.25 µm) was used in splitless mode with a programmed temperature time course (starting at 40°C and increasing, up to 250°C, at a rate of 10°C /min and beyond at a rate of 20°C /min, up to 300°C, followed by a hold time of 2 min). Helium (p = 14.5 kPa) was used as carrier gas with a total flow of 5.4 ml/min and a column flow of 0.59 ml/min. The mass spectrometer worked at an ionization energy of 70 eV and a mass range from 40 to 300 m/z.

Headspace collection was performed by solid phase microextraction (SPME) with the carboxen/polydimethylsiloxane (CAR-PDMS, 75 µm) fiber (Supelco). After exposure of the fiber to the pre-heated headspace of the grains for 30 min, its contents were injected into the GC for 1 min at an injection temperature of 250°C.

Compound identification was done using the NIST library.

### Data analysis

The QMB frequency differences, *Δ*f, between the steady-state reference (air) and the recording phase was used as feature vector. The classification of the different samples was based on a discriminant analysis. We used the partial least squares discriminant analysis (PLS-DA), which is an algorithm originally developed for quantitative regression [38]. Before the application of PLS-DA, Enose data were properly auto-scaled (zero mean, unitary variance). Discrimination models have been cross-validated by the leave-one-out method in order to estimate the classification performance. Data evaluation was done using Matlab (Mathworks).

## Results

### Species identification

**Figure 1:**
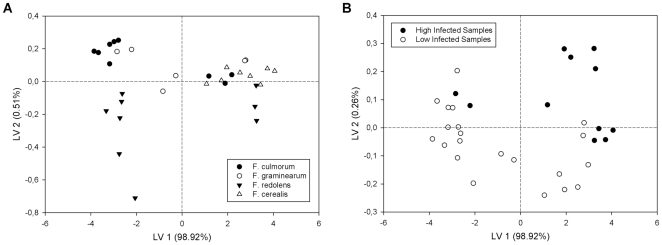
Enose results indicating different *Fusarium* species and infection levels. A: Scores plot of the first two latent variables, showing groups of *F. culmorum*, *F. graminearum*, *F. redolens* and *F. cerealis*. B: Discrimination between high- (15 days incubation) and low -infected (5 to 10 days incubation) samples of *Fusarium*. The diagram shows the scores of the first two out of 3 latent variables.

As a first step we investigated the capability of the Enose to differentiate between *Fusarium* strains. The PLS-DA model used for this discrimination in the infected samples covered five latent variables, the first two of which are shown in Figure 1A. The smallest variance within a group occured in the samples of *F. cerealis.* Samples of this group were overlapping with the highly contaminated samples of *F. redolens* and *F. culmorum.* The widest distribution was found in the samples of *F. graminearum* showing a partial overlap with *F. culmorum* and *F. cerealis.* It can be noticed that the gas chromatographic profiles from *F. redolens* and *F. culmorum* are similar. However, there was a large interclass variance detectable. Thus, the model has a satisfactory capability of differentiating different classes, as confirmed by the confusion matrix (Table 1). In the cross-validation *F. culmorum* and *F. cerealis* were perfectly recognized (100% correct). The classification rates of *F. graminearum* and *F. redolens* were 83% and 89%, respectively. The correct classification rate across all fungi was 94%.

**Table 1:** Confusion matrix of true vs. estimated values of species classification.

True value	Estimated value			
	*F. culmorum*	*F. graminearum*	*F. redolens*	*F. cerealis*
*F. culmorum*	9	0	0	0
*F. graminearum*	0	5	0	1
*F. redolens*	0	0	8	1
*F. cerealis*	0	0	0	8

Classification was done by PLS-DA.

### Classification of infection level

Correctly classifying the level of infection turned out to be slightly more difficult due to a high interspecies variability and the need for many data samples at the various concentrations. Thus, a binary classification with three latent variables in total was done (Figure 1B). For that purpose samples with an incubation time of 15 days were classified as highly infected, while samples having been incubated over 5 or 10 days were merged and classified as low-infected. This led to a higher variability in the low level probes. Nevertheless, there was a clear difference detected. Classification of the infection level (Table 2) showed that 91% of the samples were correctly classified. However, 18% of highly infected samples were classified as low-infected, all of them being *F. graminearum* samples.

**Table 2:** Confusion matrix of true vs. estimated values of binary classification of infection levels.

True values	Estimated values	
	Low	High
Low	20	1
High	2	9

Samples with incubation times of 5 to 10 days were classified as low, samples with 15 days as high. Classification was done by PLS-DA.

### Fungi vs. Control

**Figure 2:**
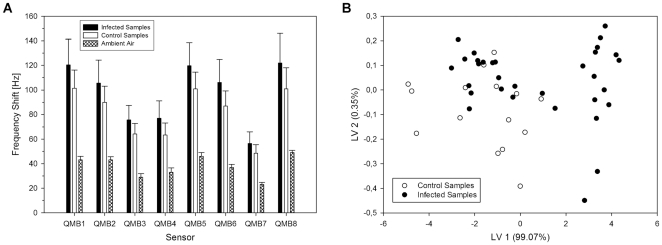
Enose results indicating *Fusarium* infection. A: Frequency shift of the quartz microbalance sensors during measurements of infected (n = 32) and control (n = 15) samples, and of ambient air (n = 5). Error bars indicate standard deviations. B: Scores plot of the first two latent variables of the PLS-DA model showing groups of infected and non-infected samples. The Model covers four latent variables in total.

For a qualitative analysis all measurements were split into two groups (infected vs. control). Figure 2A shows average sensor responses of infected vs. control samples or ambient air. For the infected samples, the responses of all sensors were on the average 16 Hz higher than for the controls. Ambient air gave very low responses. The signals were lower than 50% as compared to grain measurements. Furthermore, a PLS-DA model was performed on these data with a total of four latent variables. The scores plot of the first two variables (Figure 2B) showed a segregation of infected from control samples, although with a slight overlap. Accordingly, the major portion (91%) of infected probes are recognized correctly by cross-validation (confusion matrix, Table 3). Eight out of 47 measurements led however to a misclassification due to large variances within the groups. All of the false negatives originated from *F. redolens* at a high infection level. The variance of the *F. redolens* group might thus be explained by the fact that the high concentrations of those samples were associated with changes in the relative components of the patterns. These probes also led to false classifications during species identification (Table 1). Further, the data of the infected group showed also a rather large variance, most presumably due to the heterogeneity of the dataset consisting of probes from different fungi and at different levels of infection. In fact, using GC/MS yielded a complex composition of the headspace (Table 4). While some substances such as ethanol or hexadecane appeared in all samples, several other compounds reported to be infection-specific (2-methyl-1-propanol, 3-methyl-butanol, 1-octen-3-ol and 3-octanone [8], [23], [30], [39]) were differentially detected in some but never in all samples.

**Table 3:** Confusion matrix of true vs. estimated values, summarizing the classification results of infection.

True value	Estimated value	
	Control	Infected
Control	10	5
Infected	3	29

Estimation was done by PLS-DA classifier.

**Table 4:** Volatile compounds released by the analyzed fungi and an untreated control (sampling temperature, 70°C).

Compound	Control	*F. cerealis*	*F. graminearum*	*F. culmorum*	*F. redolens*
**Alcohols**					
Ethanol	X	X	X	X	X
2-methyl-1-propanol		X		X	X
3-methyl-butanol		X	X	X	X
1-octen-3-ol		X	X	X	X
**Carbonyls**					
2-methyl-propanal		X		X	X
Acetic Acid	X	X	X		
3-methyl-butanal		X	X	X	X
Hexanal	X	X	X	X	X
3-octanone			X		
Benzeneacetaldehyde		X			X
**Hydrocarbons**					
p-xylene			X	X	X
Hexadecane	X	X	X	X	X
3,3,4-trimethyl-hexane	X		X	X	X
3,7-dimethyl-decane	X	X	X	X	X
**Miscellaneous**					
Butyrolactone		X	X		X
2,2-dimethyl-1-propanol benzoate			X	X	

**Figure 3:**
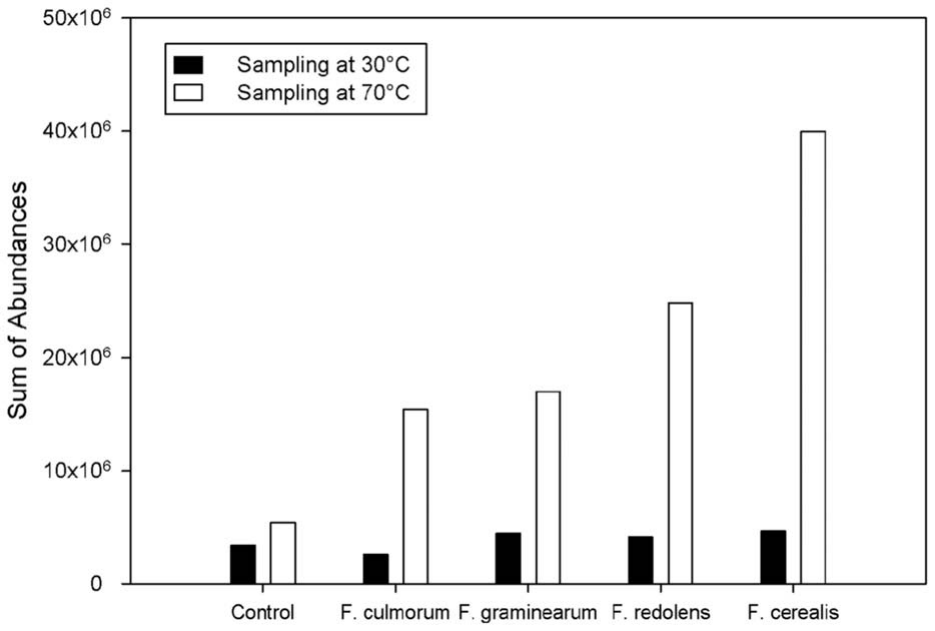
Temperature-dependency of abundancies. Comparison of total volatile abundances of four Fusaria species and controls as obtained from the GC/MS measurements taken at two different sampling temperatures.

The total amount of volatiles found in our GC/MS analysis clearly increased with increasing sampling temperature. Samples infected at 70°C emitted approximately 5-fold more volatiles than at 30°C, which expectedly led to a larger variance. In control samples the corresponding increase was just 60% (Figure 3).

## Discussion

*F. graminearum* and *F. culmorum* are known mycotoxin producers and thus potentially hazardous. It is therefore important to differentiate between *Fusarium* species. They are the most pathogenic and most frequently occurring Fusaria species [1]. In wheat and barley the pathogens lead to a destructive disease called *Fusarium* head blight (FHB). The fungi affect mainly living plants, especially during warm and wet weather conditions in the time of flowering [40]. The occurrence of FHB leads to a reduction in grain size, kernel weight, germination rate as well as a depression in quality parameters [1], [41]. Thus, high economic losses are going along with FHB infection. Furthermore there are hazardous effects of the toxic metabolites produced by *Fusarium*. Contaminated cereals, if used in livestock feed, cause a reduction in food uptake and eventually economic losses [42]. The relevance for humans should not be underestimated since mycotoxins, when entering food products, represent a potential toxicity [6].

We used a linear classifier for our data analysis. A more complex classifier such as a neural network would presumably improve the classification rate. However, there is a balance between the classifier performance and the generalisability of the results. If the complexity of the classifier is kept low, the results are usually more general, whereas results obtained with more complex classifiers tend to be less prone to generalization. For this study we therefore preferred a simple and robust classifier. The result that 18% of highly infected samples were classified as low infected ones appears to be the price for the simple classifier. As a consequence, the risk of samples classified as low infected must not be underestimated. A more complex analysis, the results of which can at the same time be generalised, is of course desirable and would certainly be preferred in many cases. We are currently developing a bionic algorithm of this kind hopefully achieving both requirements.

Our Enose analyses performed at 30°C turned out to be more reproducible than those performed at 70°C. This somewhat unexpected behavior may have the following explanations. First, while transferring the gas from the source to the sensor array, the temperature was not kept constant, so that a temperature drop might have induced condensation phenomena of some volatile compounds resulting in a change of the composition of the sample prior to entering the sensor cell. Second, the adsorption of volatiles onto a porphyrin layer is a temperature-dependent process, the efficiency of which decreases with increasing temperatures.

The false positive classifications observed are brought about by a high variance in our control groups. There are several possible reasons for this. As our model performed a binary classification, it was unable to differentiate between species or levels. Second, the control groups contained samples exposed to different incubation times. Third, incubation of non-inoculated grains may have changed the composition of odor compounds. In practical terms, false negative classifications would seriously compromise the Enose approach, while false positive classifications were considered acceptable, though unsatisfactory.

As we aimed at a qualitative recognition of infected samples, we took the classification rates as efficiency measure. The fact that all classification rates were higher than 83%, most of them being much higher, clearly indicated that chemometrical fingerprints allow the detection of fungal infection as well as the discrimination between specific Fusaria species.

In this paper we tried to bridge the obvious gap between the relevance of *Fusarium* infestation and its simple, fast, on-line, portable and quantitative measurement. We show that the metalloporphyrin-based Enose can be used to qualitatively detect and correctly classify dry, whole, *Fusarium-*infected wheat grains. Even low-contaminated grains were accurately detected, allowing them to be excluded from the food or feed chain. Second, in some practical cases a Fusarium infestation–specific signal must be recognised in the presence of a background of interfering molecular species. This would commonly be achieved–if necessary–by increasing the number of sensors. Third, the Enose meets most analytical requirements needed. It is a mobile, inexpensive and relatively fast electronic device, capable of differentiating hazardous grain from innocuous grain and thereby guaranteeing the compliance with existing health standards.
